# Supplementary material for: Recognition of Japanese university students one year after the discharge of treated water from the Fukushima Daiichi Nuclear Power Station
Source: PLoS One. 2026 Mar 10;21(3):e0344455. doi: 10.1371/journal.pone.0344455 (PMC12974853; doi:10.1371/journal.pone.0344455)
Supplement: S1 Table — (DOCX) [file pone.0344455.s001.docx]

S1: Major of the study participants

| **Major** | n | % |
| --- | --- | --- |
| **Major in Science** |  |  |
| Faculty of Medicine | 789 | 54.3 |
| Faculty of Pharmacy | 21 | 1.4 |
| Faculty of Dentistry | 15 | 1.0 |
| Faculty of Engineering | 31 | 2.1 |
| Faculty of Information and Data Science | 14 | 1.0 |
| Faculty of Environmental Science | 2 | 0.1 |
| Faculty of Fisheries | 2 | 0.1 |
| Department of Radiological Science | 243 | 16.7 |
| Department of Nursing | 23 | 1.6 |
| Department of Medical Engineering | 66 | 4.7 |
| Department of Medical Laboratory Science | 5 | 0.3 |
| **Subtotal** | 1211 | 83.3 |
| **Major in Humanities** |  |  |
| Faculty of Education | 21 | 1.4 |
| Faculty of Economics | 180 | 12.4 |
| Faculty of Global Humanity and Social Sciences | 8 | 0.5 |
| Department of Early Childhood Care and Education | 33 | 2.4 |
| **Subtotal** | 242 | 16.7 |
| **Total** | **1453** | **100.0** |
