# Supplementary material for: Recognition of Japanese university students one year after the discharge of treated water from the Fukushima Daiichi Nuclear Power Station
Source: PLoS One. 2026 Mar 10;21(3):e0344455. doi: 10.1371/journal.pone.0344455 (PMC12974853; doi:10.1371/journal.pone.0344455)
Supplement: S2 Table — (DOCX) [file pone.0344455.s002.docx]

**S2. Questionnaire English-DTW**

**Questionnaire on treated water discharged from the Tokyo Electric Power Company's Fukushima Daiichi Nuclear Power Station (FDNPS)**

1. **Sex**　1) Female 2) Male

1. **Age**【　 】years old
2. **Major in University**　【　　　　　　　　】
3. **Grade in University**

1) 1st grade 2) 2nd grade 3) 3rd grade 4) 4th grade 5) 5th grade 6) 6th grade

1. **Living areas**　【　　　　　　　　】
2. **Do you know the treated water discharged into the Pacific Ocean from FDNPS?**

1) Yes 2) No

1. **Do you believe that the Japanese government provides accurate information about the ocean discharge of treated water from the FDNPS?**

1) Yes 2) Probably yes

3) Probably no 4) No

1. **Do you accept the ocean discharge of treated water from the FDNPS?**

1) Yes 2) Probably yes

3) Probably no 4) No

1. **Do you believe that the decision-making of the Japanese public is calm and rational about the ocean discharge of treated water from the FDNPS?**

1) Yes 2) Probably yes

3) Probably no 4) No

1. **What is your most concerning impact of the discharge of treated water from the FDNPS?　(Please choose one)**

1) Impact on human health 2) Impact on the ocean environment 3) Impact on marine creatures 　4) Impact on societal issues (reputational damage, etc.) 　5) Other 【　　　】

1. **Have you ever collected information or knowledge about the discharge of treated water from the FDNPS?**

1) Yes 2) No ↓

Question for those who answered “Yes”

**How did you collect this knowledge or information? (You may select multiple answers)**

1) Lecture at a university or workshop 2) Internet 3) TV or newspaper 4)Others 【　　　】

1. **Do you believe that you can explain the differences between contaminated water and treated water?**

1) Yes 2) No

1. **Do you feel reluctant to consume kinds of seafood in Fukushima?**

1) Yes 2) Probably yes

3) Probably no 4) No
