# Supplementary material for: Recognition of Japanese university students one year after the discharge of treated water from the Fukushima Daiichi Nuclear Power Station
Source: PLoS One. 2026 Mar 10;21(3):e0344455. doi: 10.1371/journal.pone.0344455 (PMC12974853; doi:10.1371/journal.pone.0344455)
Supplement: S3 Table — (DOCX) [file pone.0344455.s003.docx]

S3. Age of the study participants

| Age | n | % |
| --- | --- | --- |
| 18 | 223 | 15.3 |
| 19 | 286 | 19.7 |
| 20 | 312 | 21.5 |
| 21 | 270 | 18.6 |
| 22 | 132 | 9.1 |
| 23 | 94 | 6.5 |
| 24 | 45 | 3.1 |
| 25 | 26 | 1.8 |
| 26 | 20 | 1.4 |
| 27 | 8 | 0.6 |
| 28 | 9 | 0.6 |
| 29 | 6 | 0.4 |
| 30 | 6 | 0.4 |
| 31 | 1 | 0.1 |
| 32 | 5 | 0.3 |
| 34 | 4 | 0.3 |
| 35 | 1 | 0.1 |
| 36 | 1 | 0.1 |
| 39 | 1 | 0.1 |
| 41 | 1 | 0.1 |
| 46 | 1 | 0.1 |
| 60 | 1 | 0.1 |
|  | 1453 | 100 |
