# Supplementary material for: Recognition of Japanese university students one year after the discharge of treated water from the Fukushima Daiichi Nuclear Power Station
Source: PLoS One. 2026 Mar 10;21(3):e0344455. doi: 10.1371/journal.pone.0344455 (PMC12974853; doi:10.1371/journal.pone.0344455)
Supplement: Attachment — Submitted filename: Reviewer.docx [file pone.0344455.s005.docx]

Thank you for your valuable comments and suggestions.

Based on reviewers' suggestions, we have revised our manuscript accordingly. Our point-by-point responses are as follows:

Reviewer #1: Overall Assessment:
This manuscript presents a timely and socially important study on the awareness and acceptance of the discharge of treated water (DTW) from the Fukushima Daiichi Nuclear Power Station (FDNPS) among Japanese university students, one year after the discharge began. Based on responses from 1,453 students, the analysis is generally sound and offers valuable insight into how information sources, trust, and demographic factors influence DTW acceptance. However, several revisions are necessary to improve the manuscript’s clarity, methodological transparency, and interpretative strength.

We appreciate your positive feedback and valuable suggestions.

Major Comments:
1. Clarification of Novelty and Significance (Introduction):
The manuscript would benefit from a clearer articulation of its novelty, particularly the rationale for focusing on the younger generation. Since this group will bear long-term responsibility for national and environmental policy, prior literature on youth engagement in environmental issues or risk communication should be cited to frame the importance of their perceptions.

Thank you for your advice. In line with your suggestion, I have added previous studies on youth engagement in environmental issues or risk communication to the introduction session (L.56-62).

2. Sampling Bias and Generalizability (Methods)
The authors state that the sample was drawn from four universities, but more detail is needed about potential sampling bias—especially with respect to students’ academic backgrounds. For example, students in medicine, nursing, or engineering may have greater baseline knowledge of radiation, which could influence their acceptance of DTW.

I appreciate your advice. Following your suggestions, I have added students’ academic backgrounds to Appendix 1 and the analysis.

In addition, please provide more detailed information about the geographic location of the universities outside Fukushima Prefecture. Since the treated water is being discharged into the Pacific Ocean, perceptions may differ between coastal areas facing the Pacific and more inland regions. Without this information, it is difficult to fully assess the effect of regional proximity to the discharge site on participants’ responses.

Furthermore, based on your suggestion, I added general information regarding the geographic distribution of universities outside Fukushima Prefecture to the Study participants of the Methods section (L.71-73).

3. Logistic Regression Model Interpretation:
In Table 3, sex was statistically significant in the chi-square analysis but not in the logistic regression model. This inconsistency should be discussed in the manuscript—perhaps in terms of confounding variables or multicollinearity.

Thank you for your advice. Based on your suggestion, we added an illustration that sex was not significant in the logistic regression model in the results( L169-170 and 215-217).

Moreover, the current model omits potentially important predictors such as residential area (Fukushima vs. non-Fukushima) and age, both of which may shape attitudes toward DTW. Including these variables in the regression model may enhance its explanatory power.

Thank you for your advice. Although area (Fukushima vs. non-Fukushima) and age appeared to be factors influencing acceptance of DTW, the chi-square test comparing acceptance vs non-acceptance did not show any significant differences, so we did not include them in the logistic model. As a trial, I constructed a model including these two factors, but no significant associations were observed. Please refer to the table below (next page) for details.

If the data are available, it would also be helpful to incorporate students’ academic discipline (e.g., science vs. humanities) into the model. Educational background may influence scientific literacy and thus affect DTW perception and acceptance.

We greatly appreciate your valuable advice. Based on your suggestion, upon incorporating Major(academic background) into the model, a significant association was identified. Consequently, the findings have been adapted to the paper.

Table 3. Independent factors associated with acceptance of DTW from FDNPS

|  |  | OR (95% CI) | *p*-value |
| --- | --- | --- | --- |
| Sex | Female/Male | 0.897 (0.648–1.242) | 0.512 |
| Age | < 21/≥ 21 | 0.989 (0.933–1.047) | 0.697 |
| Living area | Inside/Outside | 0.988 (0.684–1.427) | 0.949 |
| Major | Science/Humanities | 1.505 (1.004–2.236) | 0.048 |
| The experience of collecting information or knowledge about the DTW | Yes/No | 1.554 (1.119–2.169) | 0.009 |
| Ability to explain the difference between contaminated water and treated water | Yes/No | 1.832 (1.319–2.808) | <0.001 |
| The accuracy of information about DTW provided by the Japanese government | Yes/No | 1.683 (1.365–2.075) | <0.001 |
| The calm decision about DTW in public opinion | Yes/No | 1.833 (1.257–2.674) | 0.002 |
| Concerns about consuming seafood from Fukushima | No/Yes | 3.742 (2.7708–5.225) | <0.001 |

*Note*. Logistic regression analysis, reference; acceptance of DTW from FDNPS; OR, odds ratio; CI, confidence interval; FDNPS, Fukushima Daiichi Nuclear Power Station; DTW, discharge of treated water

4. Transparency of Survey Instrument:
To improve the study’s transparency and reproducibility, the full questionnaire should be included—either in an appendix or as supplementary material. While the Methods section describes the survey’s structure in broad terms, access to the full set of questions would allow readers to better assess construct validity, especially for subjective items like “trust in the Japanese government” or “public calm.” Including both the original Japanese and an English translation would be ideal.

Thank you for your advice on improving the clarity of the paper. Based on your suggestions, I have added the questionnaire in both English and Japanese to Appendix 2.

5. Interpretation of Media Influence and Lecture Exposure:
The finding that lecture attendance did not significantly affect DTW acceptance requires deeper interpretation. It would be helpful for the authors to discuss whether this was due to the content, quality, or timing of the lectures.

Crucially, it is unclear whether all participants were equally exposed to lectures on DTW. If lectures were only given to some students, the observed lack of effect may reflect variation in exposure rather than ineffectiveness. This should be clarified in the Methods section, and the implications discussed in the limitations.

I sincerely appreciate your valuable advice. In this study, we were only able to confirm whether participants had attended or experienced a lecture on DTW. This has been added as a limitation section (L278-283).

Additionally, the fact that passive media sources (TV/newspapers) were more strongly associated with DTW acceptance suggests that mass communication may be more effective than formal educational interventions. This has important implications for public outreach strategies.

We sincerely appreciate your valuable insights. The points you provided have been added to the discussion session (L229-232).

Minor Comments:
Terminology Consistency:
The terms "contaminated water" and "treated water" appear to be used interchangeably at times. Please clearly define both terms early in the manuscript and ensure consistent usage, given the public sensitivity surrounding their distinction.

Thank you for your creative advice. Based on your suggestions, we have added a clear definition of both terms in the Introduction session (L37-41).

Reviewer #2:

 Keywords require revision.
The respondents of questionnaire are all college students. It can not be a good representative for younger generation, because a generation includes both illiterate and literate people or consists of a wide range of literacy level. It seems the comparison between younger and older people is based on 21 years old. This is not what we understand from the title, abstract, or conclusion.

Thank you for your valuable comment. Based on your suggestion, we revised the keywords and the expression “the younger generation” to “University student”. Furthermore, we add “in Japan” because Japan’s literacy rate is close to 100%.

Line 259 can not be verified by the data in manuscript. Obviously, younger people in general has relatively less concerns about their health in comparison with older people. I wonder what are are the perceptions of people with 30 years old or higher?

Thank you for your insightful comment. As you pointed out, younger people in general have relatively fewer concerns about their health compared to older people. However, since this study focused on university students, the population aged 30 and above accounted for only about 2% of the participants (kindly refer to Appendix 2), which posed limitations for the analysis.

Manuscript requires proper illustrations to reflect their results. Tables 2 and 3 have the potential for be in box-plot figures.

Thank you for your valuable comments. Table 2 presents the results of the chi-square test, and Table 3 shows the logistic regression analysis result. Although it is possible to create a box-plot figure for Table 2, the purpose of Table 2 is to demonstrate that there is no difference in the proportions of 'Accept' and 'Do not Accept' depending on the source of DTW information, rather than to indicate differences in median or mean values. Furthermore, Table 3 cannot be converted into a figure. We apologize for not being able to fully follow your creative advice.

Authors must demonstrate the results of all required statistical analysis: ANOVA or Mann-whithney, Mendel test, alpha cronbach, Pearson regression, etc. The current manuscript is more like a Poll than a scientific paper with professional scientific analysis.

Thank you for your creative advice. This study compares results based on proportions rather than medians or means. For analyzing associations, we employed logistic regression instead of correlation analysis. This paper presents a simple approach based on the questionnaire data obtained; therefore, we did not adopt approaches that present the same results through multiple figures or analyses. This is the limitation of our questionnaire. We regret that we were unable to fully implement your suggestion.

I wonder what other independent factors, like being employed, having families, and wealth are effective on the acceptance on wastewater discharge? Can they represent how much conservative or ignorant are people to this event? In the current format, I can not approve the validity of results, unless authors improve and verify their findings with solid methodology and statistical analysis. This manuscript raises more questions than answering to the ambiguities. Therefore, it requires major revision.

Thank you for your advice. Since this questionnaire targeted university students, basic attributes such as being employed, having families, and wealth were not collected. Regarding the items you pointed out, we plan to revise the participants and the questionnaire and clarify them in the next survey with the new questionnaire. Thank you also for your suggestions concerning the future direction of the study. Based on your suggestion, we added information regarding basic attributes in the limitation section.
