# Supplementary material for: Recognition of Japanese university students one year after the discharge of treated water from the Fukushima Daiichi Nuclear Power Station
Source: PLoS One. 2026 Mar 10;21(3):e0344455. doi: 10.1371/journal.pone.0344455 (PMC12974853; doi:10.1371/journal.pone.0344455)
Supplement: Attachment — Submitted filename: Response to Reviewers.docx [file pone.0344455.s006.docx]

Thank you for your valuable comments and suggestions.

Based on reviewers' suggestions, we have revised our manuscript accordingly. Our point-by-point responses are as follows:

Reviewer #2: Regarding the revised manuscript, response letter, and clarified method and results by responses, I can now admit that this manuscript has lack of scientific results for publication:
*1- This manuscript is limited to a very specific society (university students) in Japan. This group under survey has very limited variety in social characteristics (age, job, literacy, families, etc.). The independent variables are limited to >21 or <21 age, their majors, and methods for information collecting. This group can not represent any other similar groups in Japan or other countries. Obviously, the results are not suitable with scientific perspective, as these outcomes can not be extended to any other case, group, or society.*

We appreciate your thoughtful comments regarding the generalizability of our study. As you pointed out, the present research targeted university students in Japan, who represent a relatively homogeneous population in terms of age and social characteristics. We fully acknowledge that this sampling frame limits the extent to which our findings can be generalized to other populations.

In this study, we specifically aimed to examine the perceptions of Japanese university students, who represent the next generation in a country currently DTW into the ocean. As described in the *Limitations* section, there is a potential sampling bias because the participants were drawn from two universities inside Fukushima Prefecture and two universities outside the prefecture (p. 19, L266-267).

At present, we do not have comparable data from university students or other populations in other countries; therefore, we cannot make direct cross-national comparisons. Acceptance of DTW may differ across cultural or societal contexts. In response to your comment, we have added this point explicitly to the Limitation sections of the revised manuscript (p. 19, L269-274). We believe that future research with more diverse samples, including international populations, will be essential to broaden the scientific contribution of this line of research.

We thank you again for this valuable suggestion, which has helped us to clarify the scope and limitations of our study.

*2- As explained by authors, the method for analyzing the outcomes is limited to some comparisons by percentage (%) like line 128-153. In other words, the statistical analysis was more focused on frequency (like a poll), while authors could briefly explain these outcomes by illustrations.*

We appreciate the valuable comment. The section described in lines 128–153 refers to the “Comparisons of each factor accepting DTW from the FDNPS,” and all corresponding results are summarized in Table 1. Table 1 presents both (1) the descriptive statistics of each factor and (2) the results of the chi-square tests comparing the two group participants who accepted DTW from the FDNPS and those who did not. The analyses provided in this study rely on frequency‑based statistical comparisons.

*3- In a nutshell, the method, including surveyed group and statistical analysis, have nothing special or innovative that make the manuscript different from a poll or a typical survey. The discussion also lacks scientific descriptions about the outcomes.*

We sincerely appreciate the reviewer’s thoughtful insights regarding the methodological rigor and scientific contributions of the manuscript. Our study aims to provide empirical evidence regarding the acceptance of DTW from the FDNPS within a specific population, and thus employs a population-based survey design that is commonly used in risk perception and public acceptance research. While the method itself may not be innovative in terms of technique, it was carefully designed to obtain reliable, context‑specific evidence that has not previously been investigated in this region.

4- Authors could use new approaches in survey, conceptual modeling, or etc. to strengthen their manuscript. But, unfortunately, the current revised manuscript can not meet the demands of a world-wide replicable scientific research.

We thank the reviewer for this constructive suggestion. We acknowledge that advanced methodologies—such as conceptual modeling, structural equation modeling, or novel survey frameworks—could further enhance generalizability and allow for broader replication. At the same time, the primary objective of this study was to obtain baseline empirical data from a specific local context, where no comparable dataset existed. For this purpose, a conventional survey design was considered the most appropriate and feasible approach.

Nonetheless, following the reviewer’s recommendation, we have added a paragraph in the Limitations section explicitly addressing the scope of the methodology and outlining potential future directions, including the incorporation of conceptual modeling, longitudinal follow-ups, and expanded sampling strategies to enhance international replicability (p. 21, L305-312). We appreciate the reviewer’s perspective and believe these additions improve the transparency and future applicability of the research.

Reviewer #3: The manuscript reports on university students’ awareness of the release of ALPS-treated water from the Fukushima Daiichi Nuclear Power Plant. As this is a revised version, the following comments focus on the authors’ responses to the reviewers’ comments.
The authors have provided complete and appropriate responses to the comments raised by Reviewer #1. With respect to Reviewer #2, the authors have adequately addressed the major criticisms by acknowledging the study’s limitations and revising the title accordingly.
Although Reviewer #2 characterized the study as resembling a poll, the analysis is clearly focused on university students, a population with sufficient prior knowledge and intellectual maturity to engage with the topic. From this perspective, the study does not appear to be a simple poll. While expanding the target population to include the general public could be valuable, this point can reasonably be considered a topic for future research, as noted by the authors. The awareness of university students, who are expected to play leading roles in the next generation, represents a meaningful and justified focus.

We sincerely appreciate your careful review of our revised manuscript and your constructive comments.

Reviewer #4: General comment
This study focuses on acceptance of DTW, mainly in terms of its scientific safety. However, there is another critical argument on fair and transparent decision-making process of DTW with stakeholders, including fishery sector. Though the questionnaire includes a question for “calm decision by public”, but no question is asked for acceptance of policy decision making process nor access to information on procedures of governmental decision making, conflicts with local stakeholders, etc. The paper might justify DTW, by supportive opinion by young generation, who may not be informed about conflicts with stakeholders, as such aspect is (intentionally or un-intentionally) excluded from the questionnaire. In the view of the reviewer, more essential point of DTW is lack of stakeholder involvement in decision making process, rather than scientific safety. The questionnaire could have been designed to include acceptance not only of scientific safety, but also social fairness, international justice, etc.

We sincerely appreciate the reviewer’s insightful and important comments regarding the decision‑making processes surrounding DTW and the role of stakeholder involvement. We fully agree that fair, transparent, and participatory decision‑making—particularly with stakeholders such as the fishery sector—is a critical dimension of the DTW issue, and that scientific safety represents only one aspect of broader public acceptance.

We acknowledge that the present questionnaire primarily focused on perceptions related to the scientific safety of DTW. Although one item addressed “the calm decision about DTW in public,” the survey did not include explicit questions about acceptance of the governmental decision‑making process, access to information, conflict with local stakeholders, or views on procedural fairness and justice. We agree that these aspects represent essential components of social acceptance and are highly relevant to the broader policy context.

To address this valuable point, we have added a detailed explanation in the Limitations section clarifying that:

・The survey did not assess procedural fairness, stakeholder involvement, or perceived legitimacy of the decision‑making process; therefore, it reflects only one dimension of acceptance (scientific safety–related perceptions).

・Future studies should incorporate questions on social fairness, international justice, and stakeholder engagement to more comprehensively evaluate acceptance of DTW. (p. 20-21, L297-302)

We highlight this explicitly to avoid any unintended misinterpretation of the study’s scope or conclusions.

We appreciate the reviewer’s thoughtful guidance, which has helped us clarify the intended scope of the study and ensure transparent representation of its limitations.

Specific comments
1. Response to the comment on literacy by the Reviewer 2 seems to be inappropriate. Authors responded in terms of language/character literacy, the reviewer’s comment addressed scientific literacy, in my understanding.

We thank the reviewer for pointing out this misunderstanding. We acknowledge that our previous response did not fully address the reviewer’s intended meaning. The original comment was referring not to language or character literacy, but to scientific literacy, which plays an important role in how individuals interpret information related to DTW.

In the revised manuscript, we have corrected this misunderstanding and clarified that differences in scientific literacy, rather than general reading/writing literacy, may influence respondents’ understanding of technical information on radiation, environmental monitoring, and safety standards. We added this clarification in the Limitations section, and we explicitly acknowledge that our survey did not include a direct measure of scientific literacy. (p. 21, L302-305)

2. The comment#1 above is interlinked with the comment #2 by the Reviewer 1 on sampling bias. Difference in acceptance between majors (natural science/humanities) is mentioned in conclusion, but it should be noted that the percentage of respondents between these two majors (83.4% vs. 16.6%) is significantly different from total population of graduate students. Student population of humanity is much larger than student population in natural sciences, in Japan. Such bias could have been avoided by more careful sampling design. This comment is also related to general comment above. The study seems to focus too much on natural scientific aspect, and underestimates the importance of humanity.

We appreciate this important comment regarding sampling bias and the representation of academic majors. We agree that the proportion of respondents from natural sciences and humanities in our sample does not reflect the national distribution of graduate students in Japan. As the reviewer notes, students in natural sciences constitute a larger share of the overall student population. This imbalance was influenced by differences in response rates among departments, and we recognize that this may affect the generalizability of our findings.

To address this concern, we have revised the Limitations section to explicitly acknowledge the disproportionate representation of natural science majors and their potential influence on observed acceptance levels. (p. 21, L281-286).

We also clarify that the difference observed between natural science and humanities majors should be interpreted with caution, given the uneven sample distribution.

Moreover, we fully agree with the reviewer’s point that social and humanities perspectives are essential for understanding the broader societal dimensions of DTW acceptance, including ethics, social justice, procedural fairness, and stakeholder engagement. We have strengthened the Limitation to emphasize that acceptance of DTW cannot be understood solely through a natural‑science lens, and that future studies should intentionally design sampling strategies to include a more balanced representation of humanities and social‑science students, or other populations with diverse epistemological backgrounds.

We appreciate the reviewer’s insight, which has helped us clarify the scope of our study and improve transparency regarding sampling limitations.
